# Neutralization of chemokine-like factor 1, a novel C-C chemokine, protects against focal cerebral ischemia by inhibiting neutrophil infiltration via MAPK pathways in rats

**DOI:** 10.1186/1742-2094-11-112

**Published:** 2014-06-20

**Authors:** Ling Lei Kong, Zhi Yuan Wang, Ning Han, Xiao Mei Zhuang, Zhen Zhen Wang, Hua Li, Nai Hong Chen

**Affiliations:** 1 The Key Lab of Drug Metabolism and Pharmacokinetics, Beijing Institute of Pharmacology and Toxicology, 27 Taiping Road, Haidian District, Beijing 100850, China; 2 State Key Laboratory of Bioactive Substances and Functions of Natural Medicines, Department of Pharmacology, Institute of Materia Medica, Chinese Academy of Medical Sciences and Peking Union Medical College, 1 Xiannongtan Street, Xuanwu District, Beijing 100050, China; 3 The Lab of Biopharmaceutics, Beijing Institute of Pharmacology and Toxicology, Beijing 100850, China

**Keywords:** Chemokine-like factor 1, Cerebral ischemia, Inflammation, Adhesion molecular, Neutrophil infiltration, Mitogen-activated protein kinase

## Abstract

**Background:**

Inflammation plays a key role in the pathophysiology of ischemic stroke. Some proinflammatory mediators, such as cytokines and chemokines, are produced in stroke. Chemokine-like factor 1 (CKLF1), as a novel C-C chemokine, displays chemotactic activities in a wide spectrum of leukocytes and plays an important role in brain development. In previous studies, we have found that the expression of CKLF1 increased in rats after focal cerebral ischemia and treatment with the CKLF1 antagonist C19 peptide decreased the infarct size and water content. However, the role of CKLF1 in stroke is still unclear. The objective of the present study was to ascertain the possible roles and mechanism of CKLF1 in ischemic brain injury by applying anti-CKLF1 antibody.

**Methods:**

Male Sprague–Dawley rats were subjected to one-hour middle cerebral artery occlusion. Antibody to CKLF1 was applied to the right cerebral ventricle immediately after reperfusion; infarct volume and neurological score were measured at 24 and 72 hours after cerebral ischemia. RT-PCR, Western blotting and ELISA were utilized to characterize the expression of adhesion molecules, inflammatory factors and MAPK signal pathways. Immunohistochemical staining and myeloperoxidase activity was used to determine the extent of neutrophil infiltration.

**Results:**

Treatment with anti-CKLF1 antibody significantly decreased neurological score and infarct volume in a dose-dependent manner at 24 and 72 hours after cerebral ischemia. Administration with anti-CKLF1 antibody lowered the level of inflammatory factors TNF-α, IL-1β, MIP-2 and IL-8, the expression of adhesion molecules ICAM-1 and VCAM-1 in a dose-dependent manner. The results of immunohistochemical staining and detection of MPO activity indicated that anti-CKLF1 antibody inhibited neutrophil infiltration. Further studies suggested MAPK pathways associated with neutrophil infiltration in cerebral ischemia.

**Conclusions:**

Selective inhibition of CKLF1 activity significantly protects against ischemia/reperfusion injury by decreasing production of inflammatory mediators and expression of adhesion molecules, thereby reducing neutrophils recruitment to the ischemic area, possibly via inhibiting MAPK pathways. Therefore, CKLF1 may be a novel target for the treatment of stroke.

## Background

Inflammation plays an important role in the pathogenesis of ischemic stroke and other forms of ischemic brain injury. Cerebral ischemia triggers a cascade of proinflammatory molecular and cellular events, such as rapid activation of resident cells, infiltration of various types of inflammatory cells into the ischemic brain tissue, and production of proinflammatory mediators, including cytokines and chemokines [1-3].

Chemokines are small, inducible, secreted, proinflammatory cytokines that act primarily as chemoattractants and activators of granulocytes, macrophages, and other inflammatory cells [4,5]. Chemokines constitute a large family of structurally related cytokines. This family includes more than 40 members, and has been divided into four subfamilies: CXC, CC, C and CX3C. Chemokines belong to a rapidly expanding family of cytokines. Their primary function is to control the positioning of cells in tissues and to recruit leukocytes to the site of inflammation [6]. Some studies reported that levels of a variety of chemokines such as monocyte chemoattractant protein-1 (MCP-1) increased in animal models of ischemia and patients with stroke [7,8].

The chemokine-like factor 1 (CKLF1) is a novel human cytokine of the cysteine-cysteine chemokine gene family, firstly discovered through isolation from phytohemagglutinin-stimulated U937 cells and a novel functional ligand for the C-C chemokine receptor 4 [9]. CKLF1 displays chemotactic activities in a wide spectrum of leukocytes. The expression of CKLF1 is up-regulated in various inflammatory and autoimmune diseases [10]. Recent studies suggest that CKLF1 plays an important role in brain development such as migration of nerve cells [11,12].

In previous studies, we found that the expression of CKLF1 increased in rats after focal cerebral ischemia [13]. Treatment with C19, which is a C-terminal peptide of CKLF1, as an antagonist of CKLF1, decreased the infarct volume and neurological score. This suggested that CKLF1 played important roles in cerebral ischemia and may be a potential target for treatment of cerebral ischemia [14]. However, a pathological mechanism of CKLF1 in ischemic brain damage is largely unknown. In this present study, we found that neutralization of CKLF1 using anti-CKLF1 antibodies protected against focal cerebral ischemia by inhibiting neutrophil infiltration to the ischemic region via mitogen-activated protein kinase (MAPK) pathways in rats.

## Methods

### Materials

Anti-rat CKLF1 [Genebank:NM_139111.1] neutralizing antibody obtained from Peking University Center for Human Disease Genomics. Antibodies against ICAM-1 and VCAM-1 were from Santa Cruz Biotechnology (Santa Cruz, CA, USA). Anti-myeloperoxidase (MPO) antibody was purchased from Abcam (Cambridge, MA, USA). Horseradish peroxidase (HRP)-conjugated secondary antibodies were from Pierce Biotechnology (Thermo Fisher Scientific, Rockford, IL, USA). TNF-α and IL-1β ELISA kits were purchased from NeoBioscience Technology Company (Beijing, China), and MIP-2 and IL-8 ELISA kits were purchased from Westang Biotechnology (Shanghai, China). MPO detection kit was purchased from Nanjing Jiancheng Bioengineering Institute (Nanjing, China). All other chemicals and reagents were purchased from Sigma-Aldrich (St Louis, MO, USA) unless otherwise specified.

### Animals

Male Sprague–Dawley (SD) rats (age, 7 weeks; weight, 260 to 280 g) were supplied by Experimental Animal Center of Chinese Academy of Medical Sciences (Beijing, China), and housed on a 12-hour light/dark schedule in a temperature (22 ± 2°C) and humidity (<40%) controlled room with free access to food and water. All of the procedures were in accordance with the standards established in the *Guide for the Care and Use of Laboratory Animals* published by the Institute of Laboratory Animal Resources of the National Research Council (United States) and were approved by the Animal Care and Use Committee of the Peking Union Medical College and the Chinese Academy of Medical Sciences. The animals were randomly assigned into different groups according to a computer-generated randomization schedule (http://www.random.org). The assessment of measuring infarct volume and scoring neurobehavioral outcome is blinded.

### Focal brain ischemia

Transient middle cerebral artery occlusion (TMCAO) model was performed as previously described with some modifications [15]. Briefly, under 10% chloral hydrate (4 ml/kg, intraperitoneal injection), a 4-0 nylon thread, the tip of which was burned (diameter 0.36 mm), was inserted into the right common carotid artery and advanced until the origin of the right middle cerebral artery was occluded. After 60 minutes of the occlusion, the thread was removed to allow reperfusion and the animals were returned to their cages.

### Drug administration

The efficacy of anti-CKLF1 antibody in cerebral ischemia was detected by caudal vein administration and lateral ventricle injection in a preliminary experiment. Lateral ventricle injection was more effect than caudal vein administration (Additional file 1: Table S1). Therefore, we chose lateral ventricle administration in subsequent experiments to investigate the role of anti-CKLF1 antibody in cerebral ischemia. Five microliters of anti-rat CKLF1 neutralizing antibody in saline at dose of 0.1 μg, 0.5 μg or 1 μg (n = 15 in every group) that were produced in rabbits immunized with CKLF1 or normal rabbit immunoglobulin (Ig)G (1 μg, n = 15) was applied to the right cerebral ventricle immediately after reperfusion, with the needle left in place for 5 minutes thereafter. Five microliters of saline was injected in the control group (n = 15). The coordinates of the injection site were as follows: 0.8 mm posterior to the bregma, 1.5 mm lateral to the midline, and 3.5 mm depth from the dural surface, according to the atlas. The neurological scale and infarct volume were measured at 24 hours after cerebral ischemia. In all, 130 SD rats were used; 28 of the rats were removed due to death, 12 were removed for lack of neurological impairment, and 40 rats were recruited.

To investigate the long-term efficacy of anti-CKLF1 treatment, rats were randomly divided into a sham-operated group, a vehicle group, an IgG group, 0.5 μg and 1 μg anti-CKLF1 antibody-treated groups (n = 6 in every group). Saline or antibody was administrated to the animals by the intracerebroventricular route as soon as the reperfusion procedure had been initiated. The neurological scale and infarct volume were measured at 72 hours after cerebral ischemia. In all, 52 SD rats were used; 16 of the rats were removed due to death, 6 were removed for lack of neurological impairment, and 22 rats were recruited.

### Neurological function

Neurological score was taken by Longa’s five-point scale [15]. The animals without symptoms of neurological impairment or dying after the surgery were rejected and other rats were recruited.

### Infarct analysis

After indicated time points, the animals were anesthetized, brains were removed and cut into 2-mm-thick slices, with a total of six slices per animal. The slices were immersed in a 1% solution of 2, 3, 5-triphenyltetrazolium chloride (TTC) in PBS at 37°C for 30 minutes and fixed in 4% phosphate-buffered formalin. Images of the slices were obtained with a scanner and computer. The infarct area, area of contralateral corresponding structure and area of ipsilateral corresponding structure were calculated with Image J. The infarct volume was corrected to account for edema and shrinkage due to processing. The infarction area was corrected using following formula for both edema and shrinkage:

Corrected infarct area = measured infarct area + area of contralateral corresponding structure − area of ipsilateral corresponding structure [16].

The volume of infarction in each animal was obtained from the product of average slice thickness (2 mm) and sum of infarction area in all brain slices.

### Reverse transcriptase polymerase chain reaction

Total RNA from cerebral cortex was extracted with Trizol. The expression of intercellular adhesion molecule-1 (ICAM-1) mRNA or vascular cell adhesion molecule-1 (VCAM-1) mRNA was analyzed by using RT-PCR by the following primers:

ICAM-1 sense: 5′-CAGGCCACCCACCTCACAGA-3′;

ICAM-1 antisense: 5′-GGGAGCTAAAGGCACGGCAC-3′;

VCAM-1 sense: 5′-AGAGGGGGCCAAGTCCGTTC-3′;

VCAM-1 antisense: 5′-AGACCCTCGCTGGCACATGT-3′;

β-actin sense: 5′-CGGAGACGGGGTCACCCACA-3′;

β-actin antisense: 5′-AGAGAGCCTCGGGGCATCGG-3′.

The reaction condition was: 95°C for 5 minutes, then 28 cycles of 95°C for 30 seconds, 60.5°C for 30 seconds, and 72°C for 40 seconds. PCR products were applied to 1.5% agarose gel electrophoresis and photographed under ultraviolet transilluminator. The photodensitometry was analyzed with Kodak digital science Analysis System (Eastman Kodak Company, Rochester, NY, USA).

### Western blotting

The protein of cerebral cortex was prepared for Western blot analysis. Proteins were separated by electrophoresis on 15% polyacrylamide gels and transferred to polyvinylidene difluoride membranes. The membranes were blocked by 3% BSA and then incubated with primary antibodies, the followed by HRP-conjugated secondary antibody and detected with the enhanced chemiluminescence (ECL) plus detection system (Pierce Biotechnology, Rockford, IL, USA). The density of each band was quantified by using image analysis software (Science Lab 2005 Image Gauge; Tokyo, Japan).

### ELISA

Cortex was homogenized in PBS and then centrifuged 12,000 rpm for 15 minutes at 4°C and the supernatant was used for this study. The amount of TNF-α and IL-1β were determined by rat TNF-α and IL-1β ELISA kits and the amount of monocyte inflammatory protein (MIP-2) and IL-8 were determined by rat MIP-2 and IL-8 ELISA kits. The concentrations were shown as ng/g wet tissue. The optical density was determined by a microplate reader set to 450 nm.

### Immunohistochemistry staining

Immunohistochemistry staining of neutrophils was performed using paraffin-embedded brain samples of each animal that were cut and sections were deparaffinized. First, endogenous peroxidase was blocked by 3% hydrogen peroxide. After preabsorption with normal serum, sections were incubated with rabbit polyclone anti-MPO antibody (1:100) overnight at 4°C. Then the sections were overlaid for 1 hour with biotinylated goat anti-rabbit Abs, followed by avidin-biotin complex for 30 minutes. Peroxidase activity was visualized with 3, 3-diaminobenzidine as the substrate. Omission of the primary antibody served as a negative control. The numbers of neutrophils were quantified by Image J. Quantitative data were obtained from sets of four animals, with four brain sections taken per animal.

### Measurement of MPO activity

MPO activity has been used to determine quantitatively the extent of neutrophil infiltration. The right cortex was dissected and stored at −70°C until assay for MPO. The activity of MPO was determined by MPO detection kit.

### Statistic analysis

Results were expressed as mean ± SEM. Differences between data groups were compared by Student’s *t*-test for analysis of unpaired data or one-way ANOVA. Statistical significance was accepted at *P* < 0.05.

## Results

### Anti-CKLF1 antibody reduced mortality rate and neurological score

**Figure 1:**
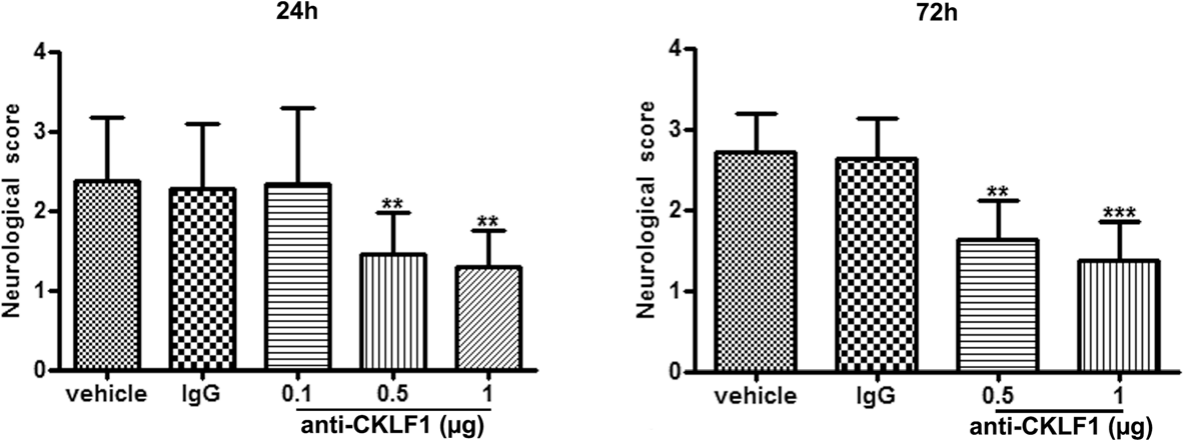
**Protective effect of anti-CKLF1 antibody on neurological function.** Bar graph shows effects of anti-CKLF1 antibody on neurological score at 24 and 72 hours after reperfusion. Anti-CKLF1 antibody (0.1, 0.5 or 1 μg) was injected into the right cerebral ventricle immediately after reperfusion. Anti-CKLF1 antibody injection improved neurological function in a dose-dependent manner at 24 and 72 hours after reperfusion. Normal rabbit IgG had no significant effect. Each data point shows the mean ± SEM for six rats. **P < 0.01, ***P < 0.001, compared with vehicle group.

Injection of anti-CKLF1 antibody significantly reduced neurological score at 24 hours and 72 hours after cerebral ischemia in a dose-dependent manner (Figure 1). The neurological score of animals treated with anti-CKLF1 antibody at dose of 0.5 μg and 1 μg was significant lower than that of vehicle group animals (*P* < 0.01); Ischemia-reperfusion caused 31.8% and 41.5% mortality in vehicle group at 24 hours and 72 hours (Additional file 2: Table S2). Pretreatment with anti-CKLF1 antibody at a dose of 1 μg decreased the mortality rate to 11.8% and 25.0% at 24 hours and 72 hours after reperfusion.

### Anti-CKLF1 antibody decreased infarct volume

**Figure 2:**
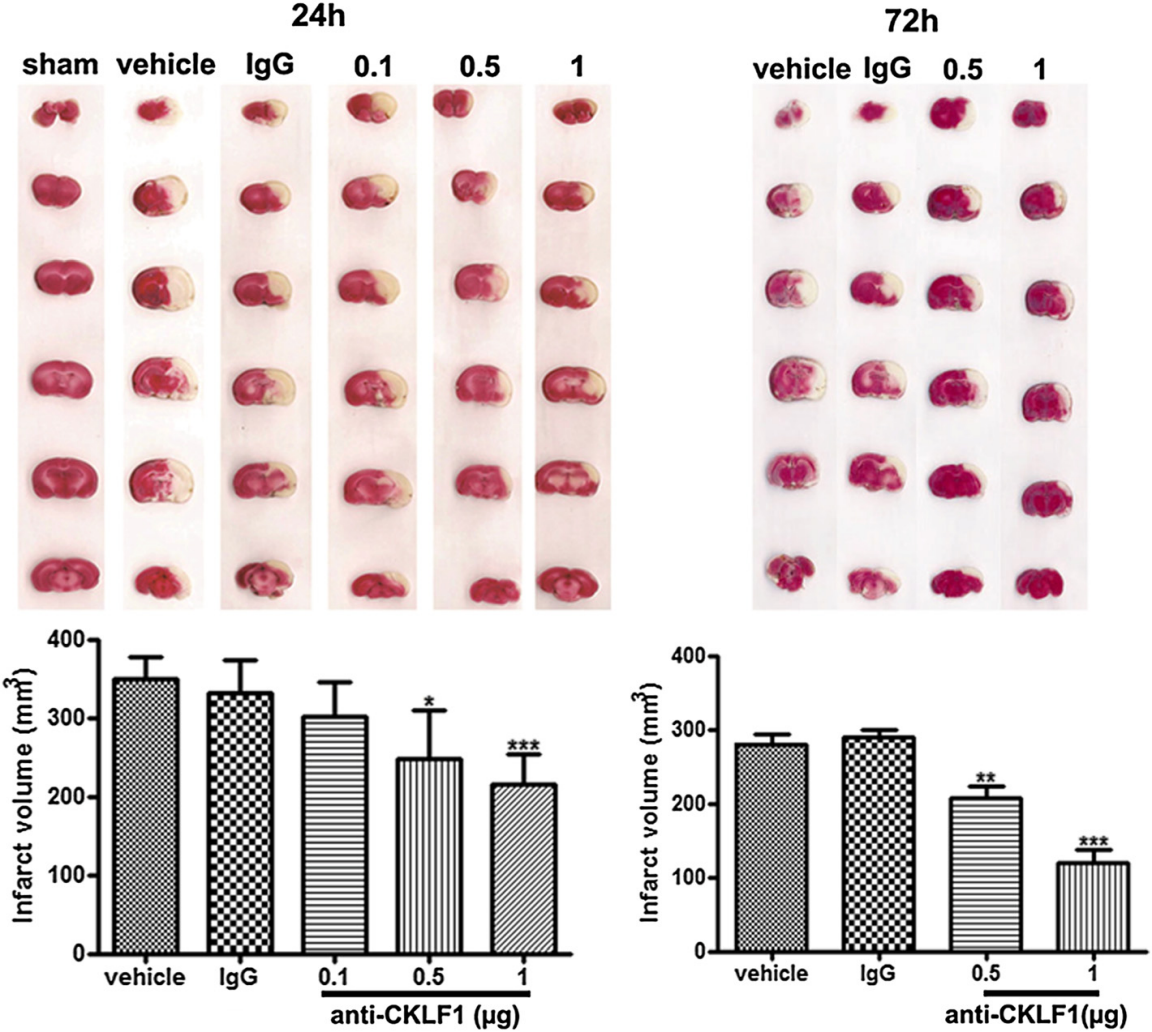
**Protective effect of anti-CKLF1 on cerebral infarct volume.** Bar graph shows effects of anti-CKLF1 antibody on infarct volume at 24 and 72 hours after reperfusion. Anti-CKLF1 antibody (0.1, 0.5 or 1 μg) was injected into the right cerebral ventricle immediately after reperfusion. The white area represents the area of infarction in the brains of ischemic rats and the non-ischemic area is red. Animals treated with anti-CKLF1 antibody showed less cerebral infarction in a dose-dependent manner compared to vehicle. Normal rabbit IgG had no significant effect. Each data point shows the mean ± SEM for six rats. *P < 0.05, **P < 0.01, ***P < 0.001, compared with vehicle group.

As shown in Figure 2, the infarct tissue was stained pale and the non-ischemic area red. Injection of anti-CKLF1 antibody could reduce infarct volume at 24 and 72 hours after cerebral ischemia in a dose-dependent manner. Anti-CKLF1 antibody at dose of 0.5 μg and 1 μg significantly reduced the infarct volume compared with vehicle group. Injection of normal rabbit IgG (1 μg) failed to reduce the infarct volume. Thus, an ameliorating effect of anti-CKLF1 antibody could be induced by specific blocking of CKLF1 activity.

### Anti-CKLF1 antibody decreased the production of TNF-α, IL-1β, MIP-2 and IL-8

**Figure 3:**
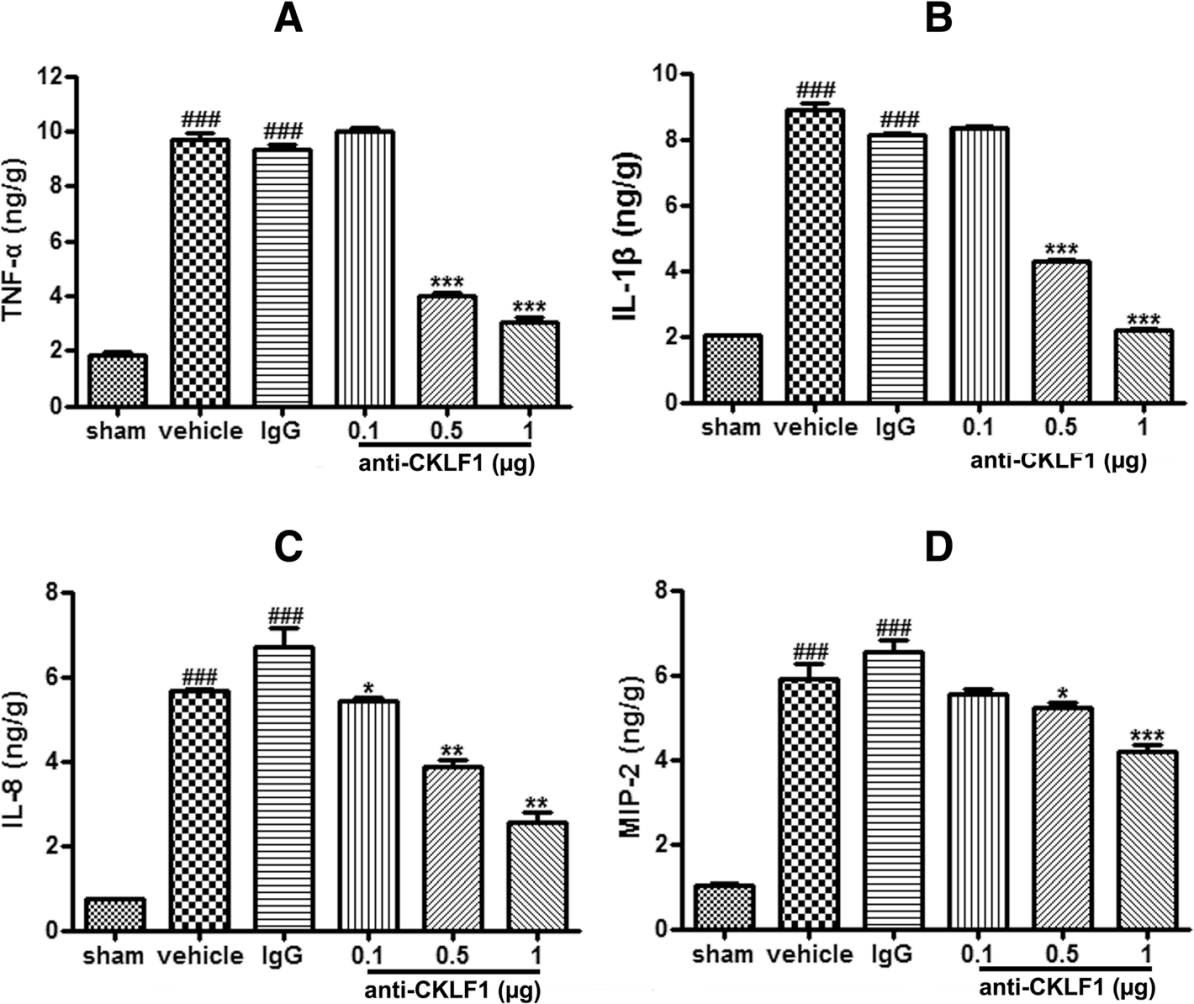
**The effects of anti-CKLF1 antibody on production of TNF-α, IL-1β, MIP-2 and IL-8.** Anti-CKLF1 antibody (0.1, 0.5 or 1 μg) was injected into the right cerebral ventricle immediately after reperfusion. Treatment with anti-CKLF1 antibody decreased the levels of TNF-α, IL-1β, MIP-2 and IL-8 in ischemic brain tissue in a dose-dependent manner. Each data point shows the mean ± SEM for five rats. ^###^*P* < 0.001, compared with sham-operated group; *P < 0.05, **P < 0.01, ***P < 0.001, compared with vehicle group. **(A)** TNF-α; **(B)** IL-1β; **(C)** IL-8; **(D)** MIP-2.

To understand the role of inflammatory factors in cerebral ischemia, the level of various cytokines and chemokines in brain was measured. As shown in Figure 3, the level of TNF-α, IL-1β, MIP-2 and IL-8 was sharply increased in vehicle group animals. Administration with anti-CKLF1 antibody lowered the amount of these inflammatory factors in a dose-dependent manner. Anti-CKLF1 antibody at dose of 0.5 μg and 1 μg could significantly decrease the level of TNF-α, IL-1β, MIP-2 and IL-8 compared with vehicle group (*P* < 0.01).

### Anti-CKLF1 antibody inhibited the expression of ICAM-1 and VCAM-1

**Figure 4:**
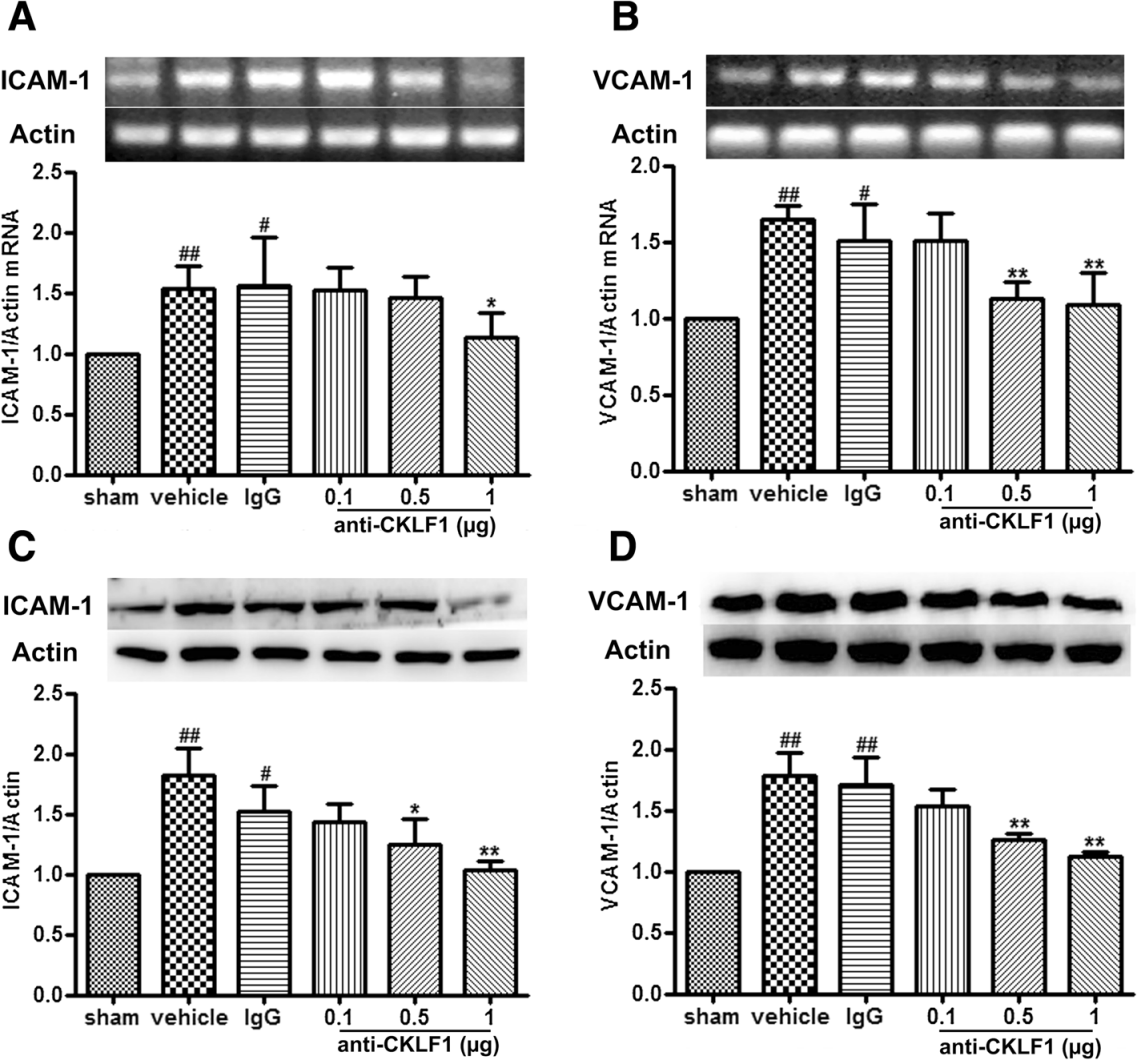
**The effects of anti-CKLF1 antibody on expression of ICAM-1 and VCAM-1.** Anti-CKLF1 antibody (0.1, 0.5 or 1 μg) was injected into the right cerebral ventricle immediately after reperfusion. ICAM-1 and VCAM-1 mRNA and protein expression were measured. Treatment with anti-CKLF1 antibody decreased the expression of ICAM-1 and VCAM-1 in ischemic brain tissue. **(A)** mRNA of ICAM-1, **(B)** mRNA of VCAM-1, **(C)** protein of ICAM-1, **(D)** protein of VCAM-1. Each data point shows the mean ± SEM for five rats. ^#^*P* < 0.05, ^##^*P* < 0.01, compared with sham-operated group; *P < 0.05, **P < 0.01 compared with vehicle group.

RT-PCR and Western blot were used to detect ICAM-1 and VCAM-1 mRNA and protein expression in the ischemic region. Marked increase of ICAM-1 and VCAM-1 mRNA (Figure 4A, B) and protein expression (Figure 4C, D) was observed in vehicle group. Treatment with anti-CKLF1 antibody at dose of 0.5 μg and 1 μg could significantly decrease the expression of ICAM-1 and VCAM-1 compared with the vehicle group (*P* < 0.01).

### Anti-CKLF1 antibody reduced neutrophil infiltration

**Figure 5:**
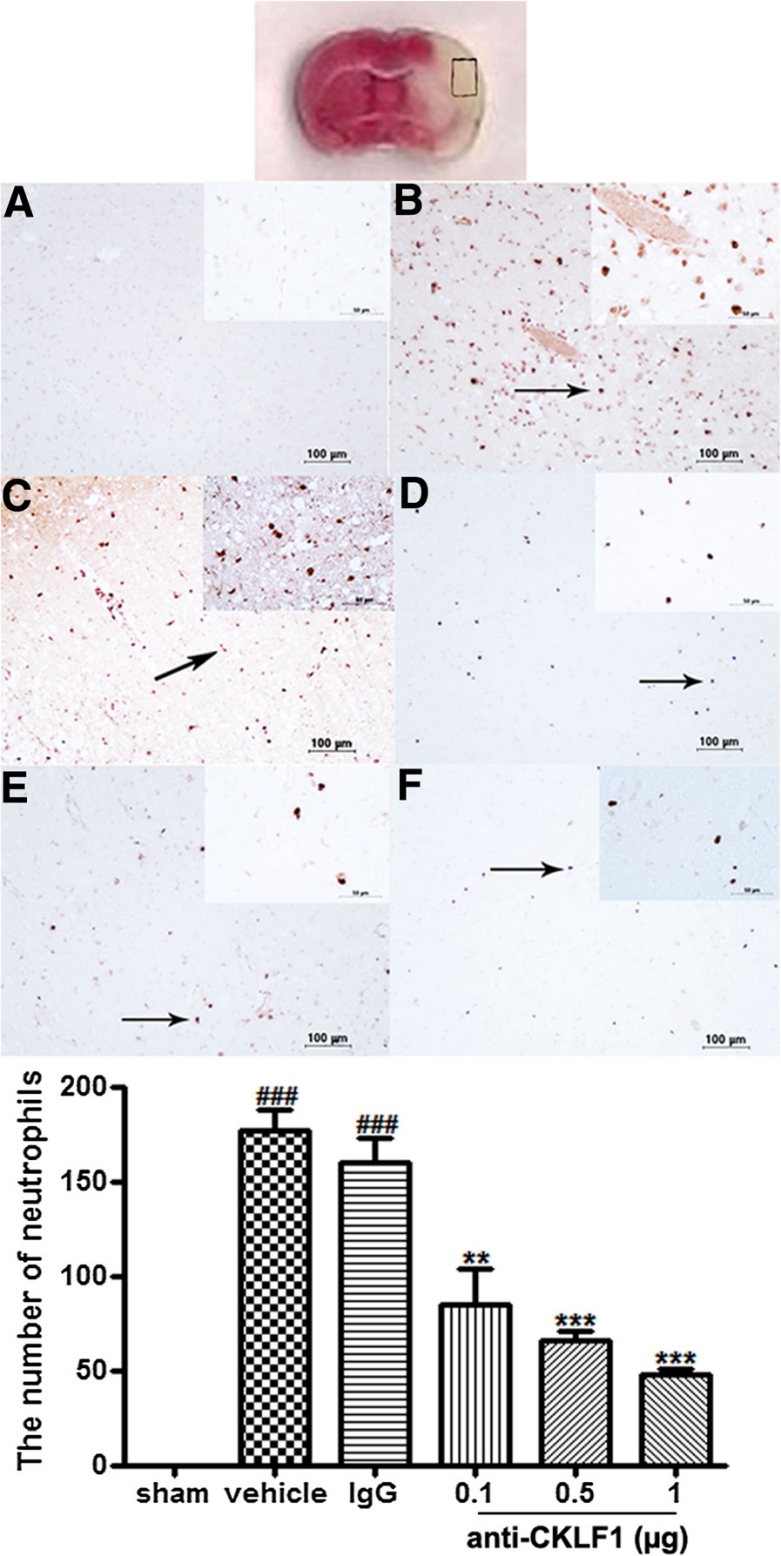
**The effect of anti-CKLF1 antibody on neutrophils infiltration.** Anti-CKLF1 antibody (0.1, 0.5 or 1 μg) was injected into the right cerebral ventricle immediately after reperfusion. Brain sections were stained with anti-MPO antibody. The MPO-positive neutrophils (indicated by arrowhead) were localized in the ischemic cortex. Treatment with anti-CKLF1 antibody decreased the numbers of MPO-positive neutrophils. The ischemic core region in cerebral cortex is indicated by pane. Cells indicated by black arrows are MPO-positive cells (brown). Each data point shows the mean ± SEM for four rats. ^###^*P* < 0.001, compared with sham-operated group; **P < 0.01, ***P < 0.001, compared with vehicle group. Typical photograph from **(A)** sham-operated group, **(B)** vehicle group, **(C)** vehicle + IgG (1 μg), **(D)** anti-CKLF1 antibody (0.1 μg), **(E)** anti-CKLF1 antibody (0.5 μg), and **(F)** anti-CKLF1 antibody (1 μg) respectively. Bar = 100 μm.

**Figure 6:**
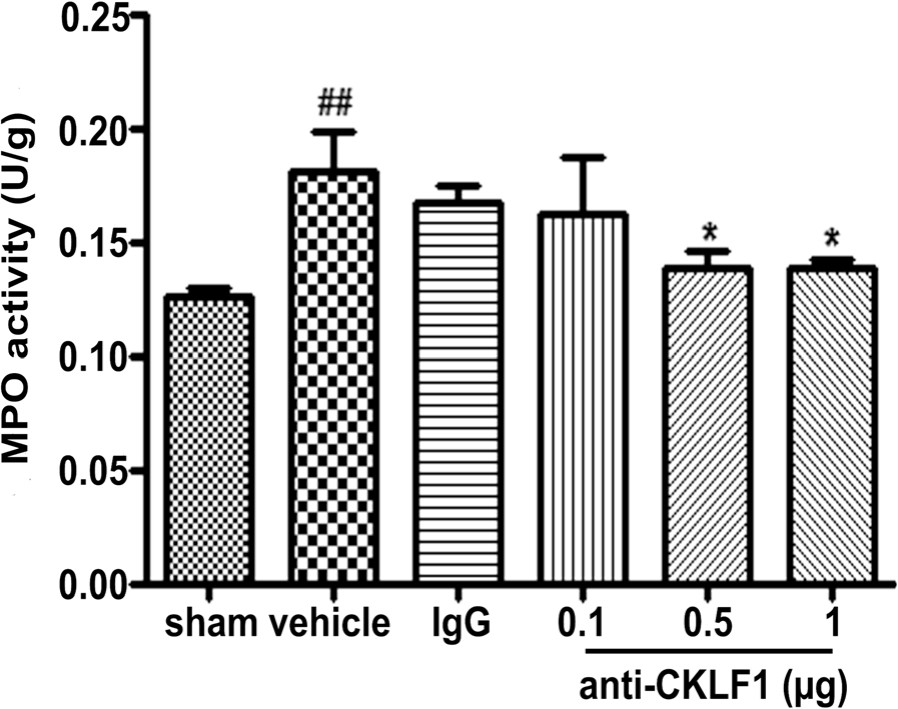
**The effect of anti-CKLF1 antibody on myeloperoxidase (MPO) activity.** Anti-CKLF1 antibody (0.1, 0.5 or 1 μg) was injected into the right cerebral ventricle immediately after reperfusion. MPO activity was measured in ischemic cortex. Treatment with anti-CKLF1 antibody inhibited the MPO activity in rat ischemic cortex. Each data point shows the mean ± SEM for five rats. ^##^*P* < 0.01, compared with sham-operated group; *P < 0.05, compared with vehicle group.

The neutrophil infiltration in ischemic and non-ischemic hemisphere was investigated by immunohistochemistry staining. The numbers of MPO-positive neutrophils were increased in the ischemic cortex 24 hours after MCAO (Figure 5B); anti-CKLF1 antibody (0.5 and 1 μg) significantly inhibited neutrophil accumulation (Figure 5E, F). In addition, the neutrophils were not observed in the contralateral hemisphere. The numbers of neutrophils were counted by Image J. A remarkable increase in neutrophils was observed in the vehicle group, and pretreatment with anti-CKLF1 antibody (1 μg) significantly reduced the numbers of neutrophils from 176 ± 11.6 to 47 ± 2.5 (*P* < 0.001). In addition, MPO activity in the brain was also measured to assess the extent of neutrophil infiltration in the ischemic region in Figure 6. MPO activity was significantly higher 24 hours after ischemia in the vehicle group than in the sham-operated group (*P* < 0.05). Treatment with anti-CKLF1 antibody resulted in decrease of neutrophil infiltration in the ischemic region.

### Anti-CKLF1 antibody inhibited the activation of MAPK signal pathways

**Figure 7:**
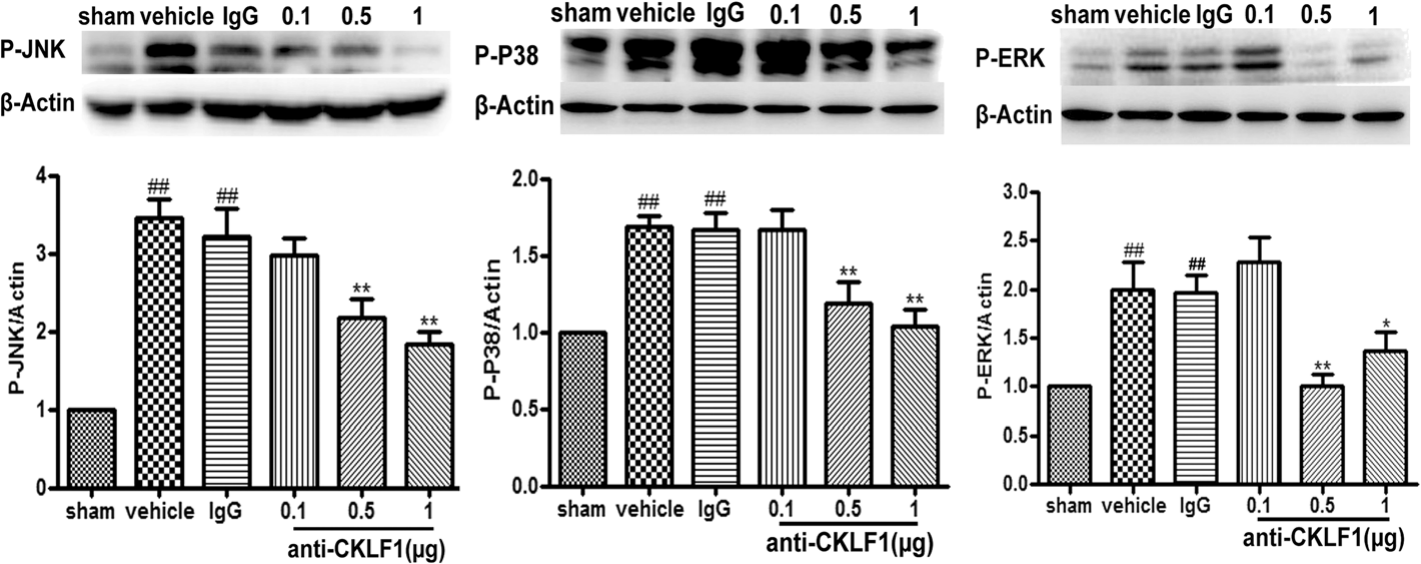
**The effect of anti-CKLF1 antibody on mitogen-activated protein kinase (MAPK) signal pathways.** Anti-CKLF1 antibody (0.1, 0.5 or 1 μg) was injected into the right cerebral ventricle immediately after reperfusion. MAPK signal proteins were measured in ischemic cortex. Treatment with anti-CKLF1 antibody inhibited the phosphorylation level of p38, ERK and JNK in the rat ischemic cortex. Each data point shows the mean ± SEM for five rats. ^##^*P* < 0.01, compared with sham-operated group; *P < 0.05, **P < 0.01, compared with vehicle group.

MAPK signal transduction pathways including p38, extracellular signal-regulated kinase (ERK) and c-Jun-N-terminal kinase (JNK) are the most important signaling molecules that are thought to mediate the inflammatory response [17-20]. Twenty-four hours after reperfusion, the phosphorylation level of p38, ERK and JNK was increased significantly in the vehicle group (Figure 7). Treatment with anti-CKLF1 antibody at dose of 0.5 μg and 1 μg could significantly decrease the phosphorylation level of p38, ERK and JNK (*P* < 0.01).

## Discussion

Our study first demonstrates that treatment with anti-CKLF1 antibody can decrease the infarct volume and improve neurological function; this is associated with inhibition of neutrophil infiltration via MAPK signal pathways.

Inflammation plays a key role in the pathophysiology of ischemic stroke [21]. Some proinflammatory mediators, such as cytokines and chemokines, are produced in human stroke and animal models of stroke and their inhibition or deficiency has been associated with reduced injury [22-26]. Therefore, the cytokines, modulating tissue injury in stroke, are potential targets in future stroke therapy [27].

As a novel member of the C-C chemokine family, CKLF1 exhibits chemotactic activity for leukocytes *in vitro* and *in vivo*. CKLF1 is up-regulated in lung tissues from asthma patients. Over-expression of CKLF1 in mice causes dramatic pathological changes in lungs that are similar to those observed in human asthma [28]. These findings imply that CKLF1 may play important roles in the inflammatory response. C19 is a C-terminal peptide of CKLF1, and contains 19 amino acids. C19 has weaker chemotactic activity and inhibits chemotaxis induced by CKLF1 or thymus and activation-regulated chemokine (CCL17) [29]. C19 can be used to treat asthma as a CKLF1 antagonist [30]. Previously, we have shown that treatment with C19 decreased the infarct size and water content [14]. However, the mechanism of CKLF1 in cerebral ischemia is unknown. In this study, we applied anti-CKLF1 antibody to assess the effects of CKLF1 on tissue injury and infiltration of inflammatory cells after cerebral ischemia in rats. In previous studies, it has been shown that neutrophils firstly infiltrated to ischemic region in various types of leukocytes and peaked at 48 to 72 hours after cerebral ischemia [21,31]. So, we investigated the protection effect of anti-CKLF1 antibody against cerebral ischemia at 24 and 72 hours after reperfusion. The data showed that treatment with anti-CKLF1 antibody decreased the infarct volume and improved neurological function at these times after reperfusion. This indicated that the protection by anti-CKLF1 antibody may be associated with inhibition of neutrophil infiltration. To validate this hypothesis, the inflammatory factors and adhesion molecules related to neutrophil infiltration were investigated.

The inflammatory factors and adhesion molecules are an important driving force in the process of neutrophil infiltration. TNF-α and IL-1β are pleiotropic cytokines released by many cell types on diverse stimulation. They exert multiple biological activities including stimulation of acute phase protein secretion and vascular permeability, and induce expression of surface adhesion molecules and other inflammatory mediators [32,33]. These actions of TNF-α and IL-1β providea central role in leukocyte infiltration and tissue injury after cerebral ischemia [34]. IL-8 and MIP-2 are CXC chemokines which are known as neutrophil chemotactic factor. Some studies have reported that neutralization of TNF-α, IL-1β, MIP-2 and IL-8 reduced neutrophils recruitment [33]. In addition, adhesion molecules on brain capillary endothelium play an important role in leukocyte migration into ischemic brain tissue. Increase of adhesion molecules expression (such as ICAM-1, VCAM-1, E-selectin and so on) has been described after stroke [32]. TNF-α and IL-1β induce adhesion molecule expression by cerebral endothelial and glial cells, thereby promoting neutrophil accumulation and migration in the microvasculature [35]. Inhibition of neutrophil infiltration into the ischemic brain via anti-adhesion molecules has been shown to reduce infarct size, cerebral edema, and neurological deficits in transient MCAO stroke models in rats and mice [36-39]. In the present study, we found that injection of anti-CKLF1 antibody decreased the production of inflammatory factors and the expression of VCAM-1 and ICAM-1, which may be associated with inhibition of neutrophil infiltration. Therefore, we also quantified the number of neutrophils in the ischemic region.

Cytokines and chemokines play crucial roles in the transendothelial migration of leukocytes such as neutrophils, monocytes/macrophages, and lymphocytes [40]. Infiltrating neutrophils amplify the cerebral inflammatory response that may exacerbate ischemic brain injury further. Most experimental and clinical studies support the importance of neutrophil infiltration in ischemic stroke [31,41]. Neutrophils adhering to the endothelium can damage the endothelium and increase the permeability of the blood–brain barrier. This leads to vascular plugging, edema and cerebral infarction [42]. Therefore, inhibition of neutrophil infiltration has a protective role in stroke. In the present study, administration with anti-CKLF1 antibody decreased the numbers of MPO-positive neutrophils and the activity of MPO, which is a marker enzyme for measuring neutrophils accumulation in the ischemic region. In addition to neutrophils, other leukocyte subsets are also involved in stroke-related inflammation. CCR4, as a functional receptor for CKLF1, is prevalently expressed on T cells and plays an essential role in the recruitment of T cells into the ischemic region in the later stages of ischemic brain injury [31]. Therefore, the role of T cells in cerebral ischemia has not been neglected. The other leukocyte subsets, including T cells, will be the next targets for the following studies to further show the effects and potential application of CKLF1.

The relationship between the MAPK pathway activation and ischemic injury have been studied and it has been demonstrated that MAPK, including phospho-p38, ERKand JNK expression, were greatly increased after permanent or temporary middle cerebral artery occlusion [43,44]. MAPK pathways may play a role in the regulation of proinflammatory cytokines during cerebral ischemia [45,46]. Some studies indicated that inhibition of the MAPK pathway reduced IL-1β expression and protected against focal cerebral ischemia [47-49]. In this study, treatment with anti-CKLF1 antibody inhibited the phosphorylation level of p38, ERK and JNK in the ischemic region. In addition, following the administration of a high dose of anti-CKLF1 antibody in healthy rats, MAPK signal pathways were not suppressed (data not shown). This may be associated with the expression level of CKLF1 in the normal adult rats. Our previous study showed that the expression of CKLF1 in the brain decreased from the embryonic stage to adulthood: the level of CKLF1 was very low in adult rats [12]. Therefore, anti-CKLF1 antibody could affect the MAPK signal pathways to inhibit neutrophil infiltration in cerebral ischemia.

Because production of inflammatory mediators and expression of adhesion molecules are both essential to neutrophil infiltration, the effect of anti-CKLF1 antibody on the production of TNF-α, IL-1β, MIP-2, IL-8, and expression of ICAM-1 and VCAM-1 may, at least in part, explain the effect on neutrophil infiltration. Therefore, the present study has indicated that anti-CKLF1 antibody attenuated the ischemic injury by inhibiting neutrophil influx into the ischemic region via MAPK pathways.

## Conclusions

This study shows that selective inhibition of CKLF1 activity significantly protects against ischemia/reperfusion injury by decreasing production of inflammatory mediators and expression of adhesion molecules, thereby reducing neutrophils recruitment to the ischemic area, possibly via inhibiting MAPK pathways. Therefore, CKLF1 may be a novel target for the treatment of stroke.

## Abbreviations

CKLF1: Chemokine-like factor 1; ECL: Enhanced chemiluminescence; ELISA: Enzyme-linked immunosorbent assay; ERK: Extracellular signal-regulated kinase; HRP: Horseradish peroxidase; ICAM-1: Intercellular cell adhesion molecule-1; Ig: Immunoglobulin; IL: Interleukin; JNK: c-Jun-N-terminal kinase; MAPK: Mitogen-activated protein kinase; MCP-1: Monocyte chemoattractant protein-1; MIP-2: Macrophage inflammatory protein; MPO: Myeloperoxidase; PBS: Phosphate-buffered saline; RT-PCR: Reverse transcriptase polymerase chain reaction; TMCAO: Transient middle cerebral artery occlusion; TNF-α: Tumor necrosis factor-α; TTC: 2,3,5-triphenyltetrazolium chloride; VCAM-1: Vascular cell adhesion molecule-1.

## Competing interests

The authors declare that they have no competing interests.

## Authors’ contributions

LLK participated in the design of the study, performed the experiments and the statistical analysis and drafted the manuscript. ZYW performed the experiments. NH performed the experiments. XMZ performed the statistical analysis. ZZW performed the experiments. HL participated in the design and helped to draft the manuscript. NHC participated in the design of study and coordination. All authors read and approved the final manuscript.

## Supplementary Material

Additional file 1: Table S1 The comparison of anti-CKLF1 antibody effect in cerebral ischemia by different administration methods. Click here for file

Additional file 2: Table S2 The effect of anti-CKLF1 antibody on the mortality rate in rats subjected to focal cerebral ischemia. Click here for file
