# Supplementary material for: Neutralization of chemokine-like factor 1, a novel C-C chemokine, protects against focal cerebral ischemia by inhibiting neutrophil infiltration via MAPK pathways in rats
Source: J Neuroinflammation. 2014 Jun 20;11:112. doi: 10.1186/1742-2094-11-112 (PMC4080607; doi:10.1186/1742-2094-11-112)
Supplement: Additional file 1: Table S1 — The comparison of anti-CKLF1 antibody effect in cerebral ischemia by different administration methods. [file 1742-2094-11-112-S1.doc]

**Additional file 1: Table S1. The comparison of anti-CKLF1 antibody effect in cerebral ischemia by different administration methods.**

| Group | Percentage of infarct volume (%) | Neurological score |
| --- | --- | --- |
| vehicle | 31.6±6.1 | 2.67±0.51 |
| Caudal vein (1mg/kg) | 25.8±4.3^#^ | 1.83±0.40^#^ |
| Lateral ventricle (1µg) | 20.2±6.3^##^ | 1.77±0.31^#^ |

Anti-CKLF1 antibody was injected by caudal vein (1mg/kg) or lateral ventricle (1µg) immediately after reperfusion. The infarct volume and neurological score were evaluated at 24 hours after reperfusion. ^#^*P*<0.05, ^##^*P*<0.01 compared with vehicle group.
