# Supplementary material for: Neutralization of chemokine-like factor 1, a novel C-C chemokine, protects against focal cerebral ischemia by inhibiting neutrophil infiltration via MAPK pathways in rats
Source: J Neuroinflammation. 2014 Jun 20;11:112. doi: 10.1186/1742-2094-11-112 (PMC4080607; doi:10.1186/1742-2094-11-112)
Supplement: Additional file 2: Table S2 — The effect of anti-CKLF1 antibody on the mortality rate in rats subjected to focal cerebral ischemia. [file 1742-2094-11-112-S2.doc]

**Additional file 2: Table S2. The effect of anti-CKLF1 antibody on the mortality rate in rats subjected to focal cerebral ischemia.**

| group | 24 h | | 72 h | |
| --- | --- | --- | --- | --- |
|  | Number of mortality/used | Mortality rate (%) | Number of mortality/used | Mortality rate (%) |
| sham | 0/15 | 0 | 0/6 | 0 |
| vehicle | 7/22 | 31.8 | 5/11 | 45.5 |
| IgG | 8/23 | 34.8 | 6/12 | 50.0 |
| Antibody-0.1 µg | 7/22 | 31.8 |  |  |
| Antibody-0.5 µg | 4/19 | 21.1 | 3/9 | 33.3 |
| Antibody-1 µg | 2/17 | 11.8 | 2/8 | 25.0 |
